# Pulmonary Fistula, Consecutive on a Scrofulous Necrosis

**Published:** 1845-12

**Authors:** 


*Pulmonary Fistula consecutive on a Scrofulous Necrosis.* By M. Grapin.—A young man, 17 years of age, but not apparently more than 12, and of scrofulous parents, when 13 years of age, became affected with scrofulous tumours and abscesses which opened in many parts of the body. On the 30th of July 1844, he was in the last degree of marasmus, with cough, purulent expectoration, and difficult breathing, with diarrhœa. Over the fifth rib of the right side, below the margin of the pectoral muscle, was a considerable sized opening, from which air and purulent matter escaped with a whistling noise during expiration. This had existed for eight months. It was previous to that time an abscess, which had opened and continued to discharge matter ever since. The rib under the opening was carious. There was no symptom of effusion within the chest or of pneumo-thorax. The chest was sonorous on percussion, excepting immediately around this aperture, where the mucous rattle was observable. On the side of the neck was a fistulous opening which communicated with the third and fourth cervical vertebræ, which were carious; œdema of the extremeties, swelling of the glands, &c. were present.

On his death it was ascertained that the opening opposite the fifth rib communicated with a sac in the lung, into which one of the larger bronchii entered. The lung at this point was adhered to the chest, and the rib was carious and fractured. In the sac in the lung was found a triangular shaped sequestrum from the rib. The interior of the cavity was lined by a shining membrane apparently continuous with the mucous membrane of the bronchus. Miliary and agglomerations of gray tubercles were found scattered throughout the lungs.—*Ibid. from Arch. Gen. de Med.*

